# *In vivo* Implantation of a Bovine-Derived Collagen Membrane Leads to Changes in the Physiological Cellular Pattern of Wound Healing by the Induction of Multinucleated Giant Cells: An Adverse Reaction?

**DOI:** 10.3389/fbioe.2018.00104

**Published:** 2018-08-14

**Authors:** Sarah Al-Maawi, Chakorn Vorakulpipat, Anna Orlowska, Tomislav A. Zrnc, Robert A. Sader, C James Kirkpatrick, Shahram Ghanaati

**Affiliations:** ^1^Department for Oral, Cranio-Maxillofacial and Facial Plastic Surgery, Frankfurt Orofacial Regenerative Medicine Lab, University Hospital Frankfurt Goethe University, Frankfurt am Main, Germany; ^2^Department of Oral and Maxillofacial Surgery, Medical University of Graz, Graz, Austria

**Keywords:** multinucleated giant cells, adverse reaction, collagen-based biomaterial, memebrane, regeneration, wound healing, integration, disintegration

## Abstract

The present study evaluated the tissue response toward a resorbable collagen membrane derived from bovine achilles tendon (test group) in comparison to physiological wound healing (control group). After subcutaneous implantation in Wistar rats over 30 days, histochemical and immunohistochemical methods elucidated the cellular inflammatory response, vascularization pattern, membrane protein and cell absorbance capacity. After 30 days, the test-group induced two different inflammatory patterns. On the membrane surface, multinucleated giant cells (MNGCs) were formed after the accumulation of CD-68-positive cells (macrophages), whereas only mononuclear cells (MNCs) were found within the membrane central region. Peri-implant vascularization was significantly enhanced after the formation of MNGCs. No vessels were found within the central region of the membrane. Physiological wound healing revealed no MNGCs at any time point. These dynamic changes in the cellular reaction and vascularization within the test-group are related typical indications of a foreign body reaction. Due to the membrane-specific porosity, mononuclear cells migrated into the central region, and the membrane maintained its integrity over 30 days by showing no breakdown or disintegration. The *ex vivo* investigation analyzed the interaction between the membrane and a blood concentrate system, liquid platelet-rich fibrin (liquid PRF), derived from human peripheral blood and consisting of platelets, leukocytes and fibrin. PRF penetrated the membrane after just 15 min. The data question the role of biomaterial-induced MNGCs as a pathological reaction and whether this is acceptable to trigger vascularization or should be considered as an adverse reaction. Therefore, further pre-clinical and clinical studies are needed to identify the types of MNGCs that are induced by clinically approved biomaterials.

## Introduction

The biomaterial physicochemical properties play a major role in induced cellular reactions (Ghanaati et al., 2012). Synthetic biomaterials are precisely synthetized under controlled conditions to produce a specific biomaterial porosity, thickness and surface topography (Moore et al., 2001). In contrast, natural biomaterials e.g., allogeneic and xenogeneic biomaterials are mostly derived from a particular region of the donor body without *de novo* synthesis (Ghanaati et al., 2011). Thereby, these materials undergo strict processing using different chemical and physical methods to reach an adequate state of purification and deactivation of potential pathogens and donor-cells, which may also affect the native structure (Ghanaati et al., 2014). In this sense, the resultant structure and surface characteristics of natural biomaterials depend on the donor tissues and the processing techniques used for their purification (Al-Maawi et al., 2017).

The type of the triggered cellular reaction in response to a biomaterial is imperative for the success of tissue engineering strategies (Dollinger et al., 2018). After biomaterial application, interaction with the surrounding host tissues and cells leads to the induction of a specific cellular inflammatory reaction that may characterize the biomaterial regeneration capacity (Ghanaati, 2012). In a complex process, the cellular reaction toward the implanted biomaterial occurs in concert with wound healing. Initially, the biomaterial surface capacity to absorb specific proteins such as fibrin is a trend-setting property for the subsequent cellular reactions and was described to be involved in the foreign body reaction (Anderson et al., 2008). The formation of a provisional matrix on the interface between the implanted biomaterial and the host tissue is the initial nexus for the host cells to interact with the biomaterial (Anderson et al., 2008). Different *in vitro* and *in vivo* models are utilized to understand the patterns of inflammatory responses to biomaterials and assess their biocompatibility and potential adverse reactions.

In the last decade, our group has presented a systematic series of *in vivo* investigations to analyze the cellular reactions toward different biomaterials using a subcutaneous implantation model (Al-Maawi et al., 2017). Basically, two types of cellular reactions were observed. A physiological reaction that includes the induction of solely mononuclear cells was observed in the case of a non-cross-linked bilayer collagen membrane leading to its integration within the host tissue (Ghanaati, 2012; Al-Maawi et al., 2017). In this *in vivo* study, the porcine-derived biomaterial maintained its structure over 60 days and showed the capacity to serve as a functional barrier without undergoing a premature breakdown or degradation. Moreover, transmembraneous vascularization was not necessary for the integration of this collagen membrane (Ghanaati, 2012). A similar reaction was detected within the implantation bed of a non-cross-linked collagen matrix made from porcine skin and peritoneum (Ghanaati et al., 2011). This biomaterial evoked only mononuclear cells over 60 days and maintained its native structure, which led to its integration within the implantation region. In addition, these findings were successfully translated to the clinic, showing the same mononuclear cell-based reaction (Ghanaati et al., 2011).

The second type of tissue response included the formation of multinucleated giant cells (MNGCs) as a pathological cellular reaction toward the biomaterials (Al-Maawi et al., 2017). The presence of MNGCs within the subcutaneous implantation bed of two non-cross-linked, collagen-based biomaterials of different thicknesses led to their disintegration in terms of premature loss of their native structure as well as an influx of the host connective tissue into the membrane after 30 days, leading to biomaterial disintegration. Thus, the cellular reaction toward biomaterials depends on their physicochemical properties (Barbeck et al., 2015a,b; Al-Maawi et al., 2017). The role of biomaterial induced MNGCs within the regeneration process is not yet fully understood. Different aspects are discussed in the literature to whether these cells may have any contribution to the regeneration process by expressing possible anti-inflammatory mediators (Miron and Bosshardt, 2018). On the other hand aliterature review has shown that biomaterial induced MNGCs actually express similar proinflammatory pattern as pathological MNGCs known from inflammatory diseases such as tubercolusis (Al-Maawi et al., 2017).

In addition to the manufacturing techniques, further methods are used to enhance the stability of collagen-based biomaterials including different types of cross-linking. However, cross linking techniques, especially chemical cross linking was shown to induce a high foreign body reaction (Rothamel et al., 2005). To avoid this, clinical techniques were introduced to provide the membrane higher biomechanical stability without cross linking. In this sense, the collagen double layer technique was introduced for enhanced membrane stability during guided bone regeneration (Abou Fadel et al., 2018). However, degradable biomaterials cannot be considered as a physically occlusive barrier as it is the case for non-resorbable biomaterials (Ghanaati, 2012). Recent studies have shown that resorbable biomaterials serve rather as a functional barrier for a defined time period and get then integrated into the implantation region (Ghanaati et al., 2011; Ghanaati, 2012).

Further developments have focused on harvesting collagen from different animal groups and compartments. Thereby, a novel collagen biomaterial with a specific structural architecture was derived from bovine achilles tendon. The aim of the present study was to analyze the *in vivo* cellular response toward this biomaterial. A subcutaneous implantation model in Wistar rats was used to analyze the cellular reaction in comparison to physiological wound healing without a biomaterial over 30 days. Special focus was placed on the inflammatory pattern, vascularization and regenerative capacity. A liquid platelet-rich fibrin (liquid PRF), which is a blood concentrate system derived from centrifuged human peripheral blood components including fibrin, leukocytes and platelets, was used to examine the protein absorption capacity and interaction with human cells as a novel *ex vivo* assessment system.

## Materials and methods

### SYMBIOS® collagen membrane SR

SYMBIOS® Collagen Membrane SR (SB, Dentsply Implants, Germany) is a slowly resorbing membrane matrix engineered from highly purified type I collagen fibers derived from bovine achilles tendon. According to the manufacturer, the harvested collagen underwent a purification and processing procedure including the use of sodium hydroxide for the inactivation of pathogens such as those associated with bovine spongiform encephalopathy (BSE). The processing and purification methods met the European and international standards for animal tissue sourcing.

### *Ex vivo* study

The *ex vivo* part of the study focused on the histological analysis of the initial biomaterial-cell interaction to assess the membrane capacity to absorb human proteins and interact with mononuclear cells from the peripheral blood.

#### Liquid platelet-rich fibrin (liquid PRF) preparation

PRF is a blood concentrate system derived from centrifuged human peripheral blood. This concentrate contains a high number of platelets and leukocytes in addition to fibrinogen and plasma proteins. Liquid PRF was chosen to mimic the initial interaction between the biomaterial and the host tissue after biomaterial application.

Three healthy volunteers between 20 and 60 years old donated blood for this study. All volunteers gave written informed consent beforehand. The liquid PRF preparation was performed as previously published (Chia-Lai et al., 2017; Wend et al., 2017). Peripheral blood was collected using 10-ml plastic tubes (orange tubes, PROCESS for PRF, France) and clinically approved butterflies. Two 10-ml tubes per donor were collected and immediately placed in a preprogramed centrifuge (DUO™, PROCESS for PRF, France). Centrifugation was performed for 8 min at 600 rpm, 44 g. The resultant upper layer (liquid PRF) was collected using a syringe with a needle (BD Microlance™ 3, Germany). Nine biomaterial samples (3 per donor) 10 × 10 mm^2^ in size were placed in a 24-well plate. One milliliter of liquid PRF was added to each biomaterial sample and incubated for 15 min. Thereafter, the samples were fixed in 4% buffered formalin for 24 h for further histological analysis.

### *In vivo* experimental design: animal surgery

The present *in vivo* protocol was approved by the committee on the Use of Live Animals in Teaching and Research (State, Darmstadt, Hessen Germany). A total number of 32 female 8-week-old Wistar rats were purchased from Charles River Laboratories (Germany) and housed for a week before use at the Animal Welfare Office and Central Facility (Goethe University, Frankfurt, Germany). The animals were randomly distributed into 2 groups (*n* = 16 animals per group). The surgical procedure followed standardized methods as previously described (Ghanaati, 2012). In brief, after intraperitoneal anesthesia (10 ml of ketamine (50 mg /ml) with 1.6 ml of 2% xylazine), the first group (*n* = 4 per time point) of animals was placed under anesthesia, and a sterile collagen membrane (SYMBIOS® Collagen Membrane SR, Dentsply Implants, Germany) was implanted under strict sterile conditions into a subcutaneous pocket within the rostral subscapular region. The second group was sham operated (*n* = 4 per time point) to analyze the cellular reaction under physiological wound healing. The animals were sacrificed by means of an overdose (ketamine and xylazine 4 times the anesthetic dose). After the evaluation time points at 3, 10, 15, and 30 days post-operation, the biomaterial including the peri-implantation region in the first group as well as the sham operated region in the second group were explanted and fixed in 4% buffered formalin for 24 h for further histological preparation.

### Tissue preparation for histology and immunohistochemistry

The histological processing and staining procedures were performed as previously described (Ghanaati, 2012; Barbeck et al., 2016). Briefly, the explants were cut into three identical segments, which included the margins and the center of the implantation area. Subsequently, the *ex vivo* and *in vivo* samples were processed using a series of graded alcohol and xylene followed by paraffin embedding. For the histological and immunohistochemical staining, four consecutive 3–4-μm slices from the central segment were cut using a rotation microtome (Rotationsmicrotom RM2255, Leica, Germany). After deparaffinization and rehydration, histochemical staining of the *in vivo* and *ex vivo* samples included Mayer's hematoxylin and eosin (H and E), Azan stain and Masson-Goldner stain. To identify tartrate-resistant acid phosphatase (TRAP) activity of the cells, specific staining was performed for TRAP as previously described (Ghanaati et al., 2013). A Sample from previous (Ghanaati et al., 2010a) study showing TRAP-positive cells in Wistar rats served as a positive control for TRAP-staining (data not shown). Two more slices of the *in vivo* samples were used for further immunohistochemical staining to determine blood vessel density and identify macrophages. Immunohistochemical staining was performed using a Lab Vision^TM^ Autostainer 360-2D instrument (ThermoFisher Scientific, Germany) as previously described (Barbeck et al., 2016). Next, endogenous peroxidase activity was blocked using 4% H_2_O_2_ in methanol, and endogenous avidin- and biotin-binding proteins were blocked by avidin and biotin blocking solutions (Avidin/Biotin Blocking Kit, Vector Laboratories, USA). The first antibody anti CD-68 (MCA341GA; 1:400; 30 min) for macrophages and Anti-Actin, α-Smooth Muscle (SMA) A5228; 1:1,000; 2 h) for vascular endothelial cells. *Ex vivo* samples were stained using anti CD-61 (Dako; 1:50; 1 h) to stain platelets. Thereafter, the secondary antibody goat anti-rabbit IgG-B (sc-2040; 1:200, Santa Cruz Biotechnology, USA) was applied. Subsequently, the avidin-biotin-peroxidase complex (ABC, ThermoFisher Scientific, Germany) for CD-68 and the Histostain-Plus IHC Kit including AEC (ThermoFisher Scientific, Germany) for SMA were applied for 30 min and 20 min, respectively. As negative controls, immunological staining in the absence of the primary antibody was performed on 2 control sections. For visualization by light microscopy, the sections used for immunohistochemistry were counterstained with Mayer's hematoxylin.

### Qualitative histological analysis

Qualitative histopathological evaluation was performed using a Nikon ECLIPSE 80i light microscope (Nikon, Japan). The analysis focused on characterizing of the cellular reactions, inflammatory responses and vascularization. A further aim of the histological analysis was to examine the interaction of the biomaterial with liquid PRF *ex vivo*. Photomicrographs were captured using a camera DS-Fi1 (Nikon, Japan).

### Quantitative histomorphometric analysis

Quantitative histomorphometric analysis of the stained slides was performed with a light microscope (ECLIPSE 80i; Nikon, Japan) including a motorized scanning stage (ProScan III, Prior, USA) connected to a PC running NIS Elements software (Nikon, Japan). As previously described (Ghanaati, 2012; Barbeck et al., 2015a), images of the total implantation beds (total scans), large images of the sample including the collagen membrane and the peri-implant tissue, were reconstructed automatically by merging 100-130 individual micrographs. To evaluate the mean membrane thickness at each time point, the total scans of the H and E-stained slides were used. Fifteen distinct points along the length of the biomaterial per animal were measured manually using the “annotations and measurements” function of the NIS Elements software (Nikon, Tokyo, Japan). The mean of these measurements per slide was calculated as the absolute membrane thickness in μm. The values obtained from later time points were calculated as a percent, while the membrane thickness at day 3 was defined as 100% to avoid artifacts due to histological preparation. The number of MNGCs as well as CD-68-positive macrophages was counted manually using the “count” tool in NIS Elements on the total scans of the TRAP staining and CD-68 staining, respectively, in each animal. The total number of each cell type was calculated with respect to the total implant area on the slides (cell number/mm^2^) at each time point. The SMA-stained slides were used for evaluation the vascularization pattern. The number and the area (in mm^2^) of vessels within the implantation beds were determined by manually marking the vessels within the digitized scans. Thus, the total number of vessels was calculated in relation to the total area (in vessels/mm^2^) and as a percentage of the vessel area (as a fraction of the total implant area in %).

### Statistical analysis

The results from the calculated histomorphometrical analysis were evaluated for significant differences at different time points using one-way and two-way analyses of variance (ANOVA). Statistical significance was defined via *p*-values (^*^/•*p* < 0.05; ^**^/••*p* < 0.01; ^***^/•••*p* < 0.001 and ^****^/••••*p* < 0.0001) using GraphPad Prism 7 Software (La Jolla, USA). The results are presented as the mean ± standard deviation, and GraphPad Prism 7 was used to produce charts and complete the statistical analysis.

## Results

### *Ex vivo* histological analysis

**Figure 1:**
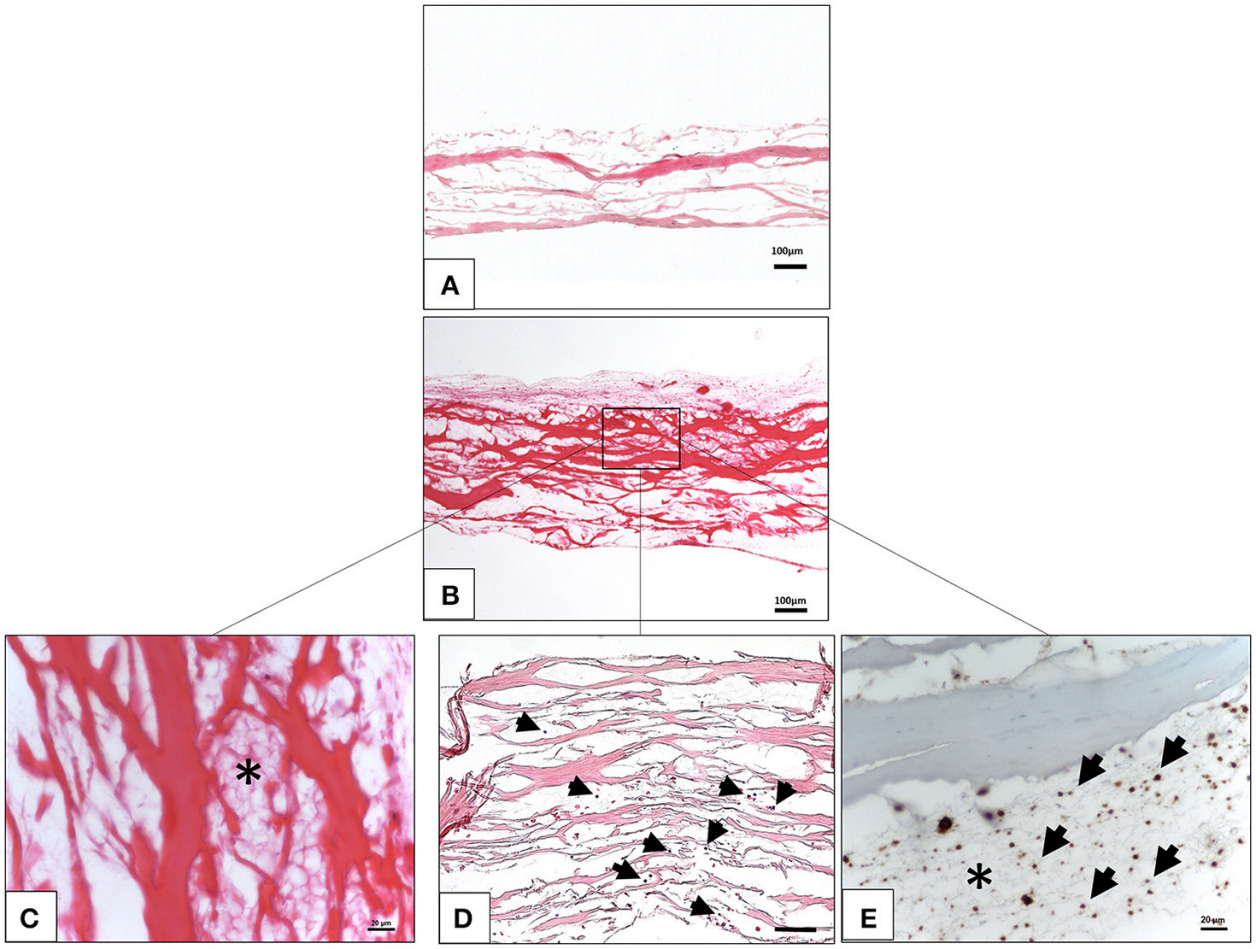
*Ex vivo* interaction between liquid platelet-rich fibrin and the collagen membrane SB. **(A)** A control of the SB illustrating the membrane-specific porous structure (H and E staining; x10 magnification; scale bar = 100 μm). **(B)** Total penetration of leukocytes and platelets from liquid PRF into the SB central region (H and E staining; x100 magnification; scale bar = 100 μm). **(C)** High magnification micrograph showing the fibrin network (^*^) within the SB collagen fibers (H and E staining; x400 magnification; scale bar = 20 μm). **(D)** High magnification micrograph showing the leukocytes (black arrows) within the SB collagen fibers (H and E staining; x200 magnification; scale bar = 100 μm). **(E)** High magnification micrograph showing the platelets (black arrows) and fibrin network (^*^) within the SB collagen fibers (anti CD-61 staining; x400 magnification; scale bar = 20 μm).

The interaction between the liquid PRF and the collagen membrane SB was analyzed histologically. The membrane showed a specific structure with differently oriented collagen fibers (Figure 1A). The membrane absorbed PRF, showing a fibrin clot within its porous structure (Figures 1B,C). In addition, leukocytes and platelets were detected within the central region of the membrane (Figures 1D,E).

### *In vivo* histological and histomorphometrical analyses

All animals survived the implantation period. The wound healing was appropriate in the test group as well as in the sham operated animals. No signs of infection or atypical feeding or sleeping behaviors were observed during the evaluation period.

#### Qualitative analysis of the cellular reaction over time

**Figure 2:**
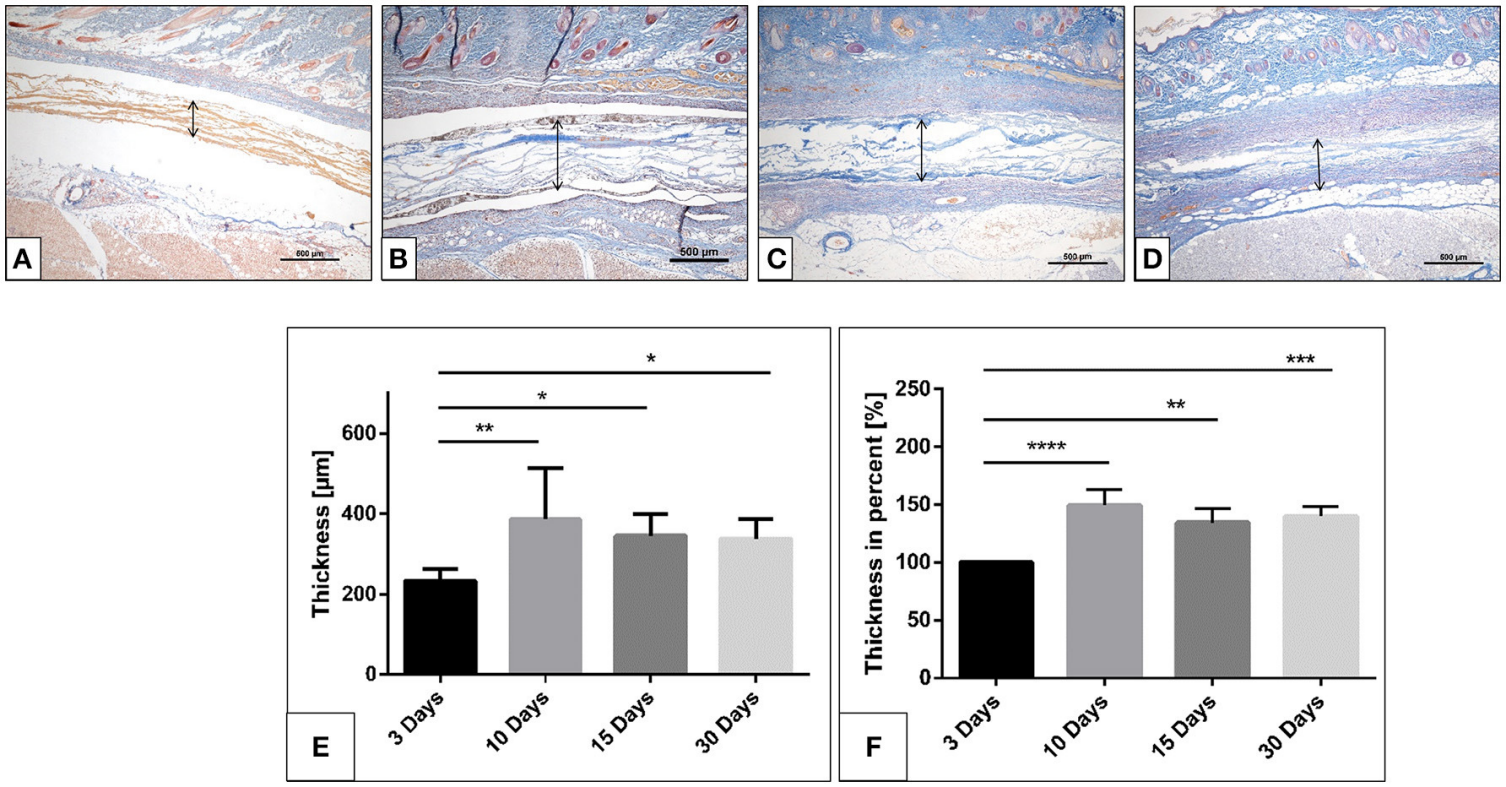
The collagen membrane SB within the implantation bed over the investigates time points. **(A)** after 3 days; **(B)** after 10 days; **(C)** after 15 days; **(D)** after 30 days. (Azan staining; x40 magnification; scale bars = 500 μm). **(E,F)** Histomorphometrical image of the membrane thickness and percentage thickness over 30 days (^*^p < 0.05; ^**^p < 0.01; ^***^p < 0.001; and ^****^p < 0.0001).

For the test group, the biomaterial was detected within the implantation region at all-time points. Three days after implantation, the membrane maintained its native structure and integrity and induced mononuclear cells, which were found on the membrane surface (Figures 2A–D). The biomaterial central region was mostly free of cells (Figure 2A).

After 10 days, the biomaterial showed a stable structure. More mononuclear cells were accumulated on both sides of the membrane. Moreover, mononuclear cells started invading the membrane and were found within the pores of the membrane (Figure 2B). CD-68-positive macrophages were accumulated on the biomaterial surface (**Figure 4A**). At this time point, single MNGCs were sporadically found within the biomaterial implantation bed and on the biomaterial surface. Most of the MNGCs showed no TRAP activity (data not shown). Additionally, micro-vessels were detected in proximity to the biomaterial. However, no vessels were detected within the membrane central region.

**Figure 3:**
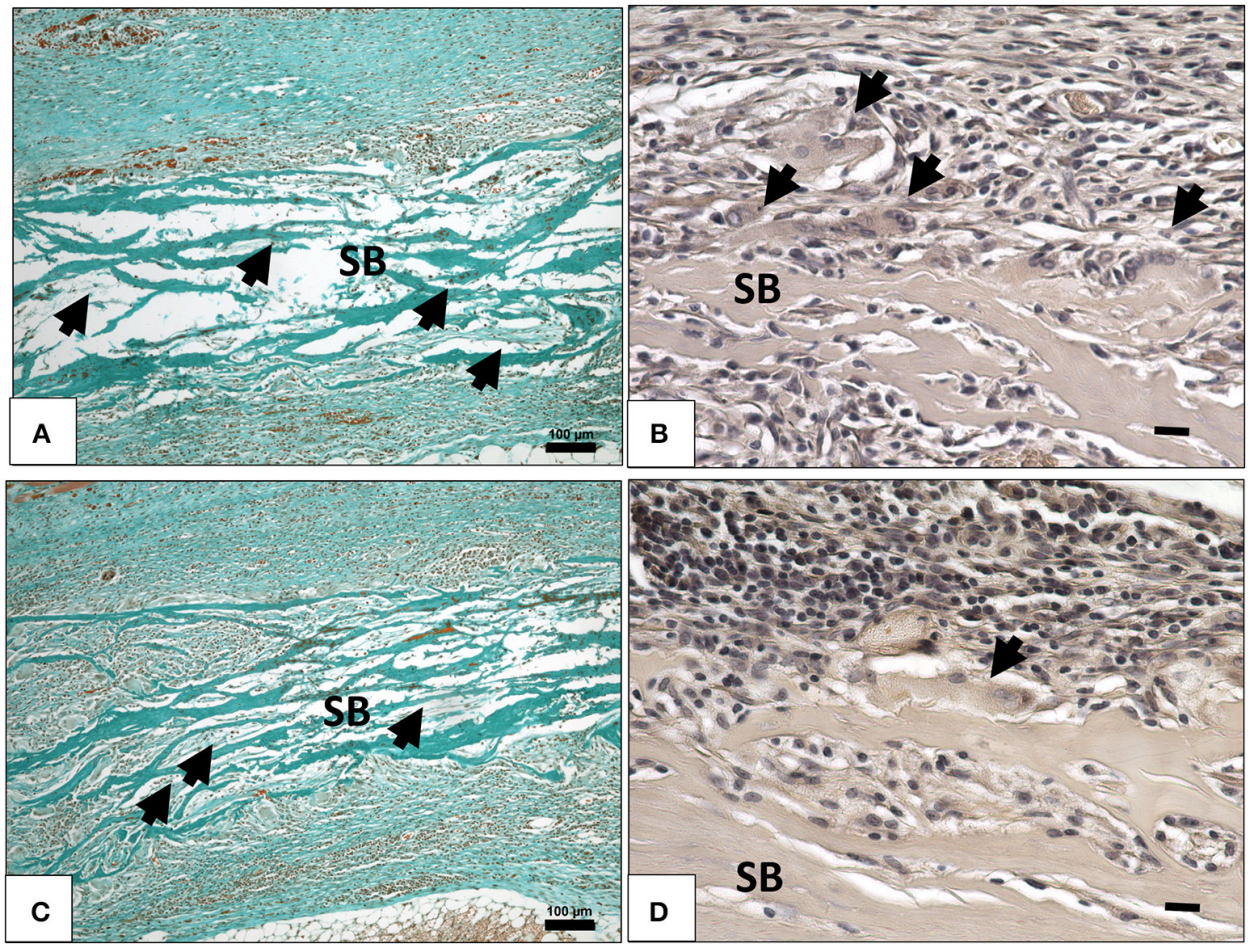
**(A)** Cellular and connective tissue infiltration (black arrows) of the collagen membrane (SB) on day 15, (Masson Goldner staining; x100 magnification; scale bar = 100 μm). **(B)** TRAP- negative MNGCs (black arrows) on the membrane surface (SB) on day 15, (TRAP staining; x400 magnification; scale bar = 20 μm). **(C)** Cellular and connective tissue infiltration (black arrows) of the collagen membrane (SB) on day 30, (Masson Goldner staining; x100 magnification; scale bar = 100 μm). **(D)** TRAP-negative MNGCs (black arrows) on the membrane surface (SB) on day 30, (TRAP staining; x400 magnification; scale bar = 20 μm).

**Figure 4:**
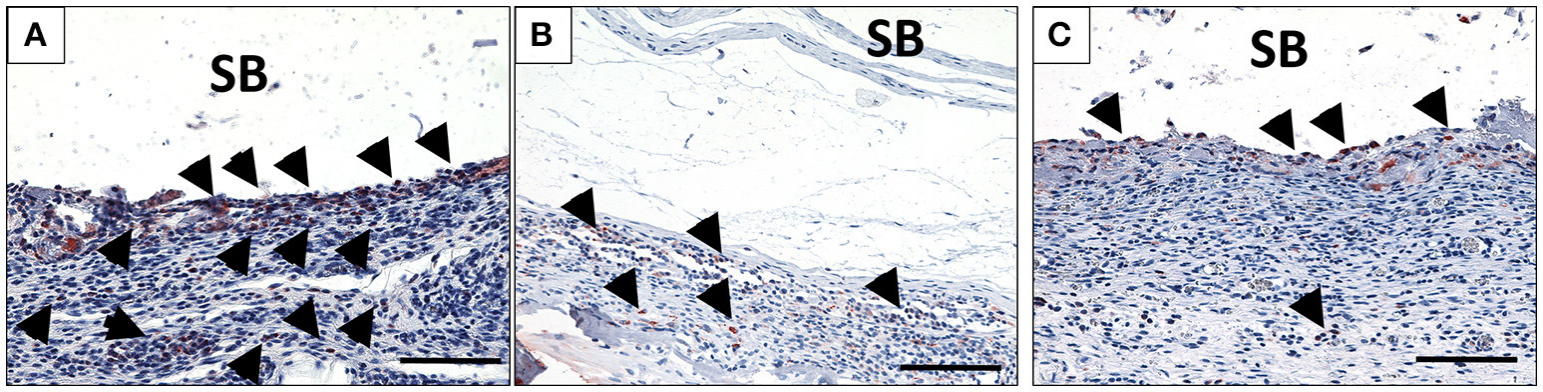
The behavior of macrophage (black arrows) accumulation on days 10 **(A)**, 15 **(B)**, and 30 **(C)** on the surface of the biomaterial (CD-68 immunohistochemical staining; x200 magnification; scale bar = 100 μm).

Fifteen days following implantation, the membrane maintained its integrity and showed stable structure. No signs of breakdown were observed. The membrane was embedded in a cell- and vessel-rich connective tissue. At this time point, more mononuclear cells invaded the membrane and reached its central region. The membrane interfibrillar area contained connective tissue (Figures 3A,B). The number of MNGCs increased remarkably, while fewer CD-68-positive macrophages were found in proximity to the biomaterial (Figure 4B). However, in general, the MNGCs showed no TRAP expression. The implantation bed showed higher vascularization at 15 days than at the previous time point. However, no vessels were found within the membrane central region (Figures 2D, 3A,B).

At 30 days after implantation, the membrane was detectable within the implantation bed, showing a stable volume and sustained integrity. No signs of breakdown or disintegration were revealed. The membrane was embedded in a cell- and vessel-rich host connective tissue and allowed the infiltration of mononuclear cells and connective tissue into its central region without losing its native structure (Figure 3D). Similar to the observations at day 10, some CD-68-positive macrophages were observed close to the biomaterial surface (Figure 4C). However, the MNGCs persisted but were not increased in number and were located on the membrane surface. Thus, no infiltration of MNGCs within the central region was observed. At this time point, most of the MNGCs showed no TRAP activity (Figure 3D). The implantation area showed new, well-vascularized connective tissue, while the biomaterial retained its native structure and included the newly formed connective tissue (Figures 2E, 3C,D).

In the control group, the physiological wound healing was uneventful during the observation period. The cellular reaction included only mononuclear cells. No MNGCs were observed at any time point. At day 3, a high number of macrophages (CD-68-positive) was observed within the evaluation area. The number of macrophages decreased progressively toward day 30. A mild vascularization pattern was detected in the healing area with an increasing tendency from day 3 to day 30.

### Quantitative histomorphometrical analysis

#### Evaluation of the membrane thickness over time

The membrane maintained its stable structure over the observation time period. The histomorphometrical analysis showed that on day 3 the membrane exhibited a mean thickness of 233.5 ± 29.4 μm. The mean thickness increased toward day 10 (366.4 ± 104.8 μm). The membrane thickness at day 15 (345.3 ± 54.2 μm) was comparable to that at day 10. Thus, no statistically significant difference was detected between day 15 and day 10. Finally, a similar value was measured on day 30 (325.7 ± 51.3 μm). The statistical analysis showed a significant difference in the membrane thickness on day 3 and those measured on days 10 (^**^p < 0.01), 15 (^*^p < 0.05) and 30 (^*^p < 0.05). However, no statistically significant differences were found among the thicknesses measured on days 10, 15, and 30 (Figure 2C).

The analysis of the percent thickness in relation to day 3 showed a similar pattern. Thereby, the mean percent thickness increased after 10 days (149.2 ± 13.5%). The membrane maintained the percent thickness on days 15 (134.5 ± 12.1%) and 30 (139.9 ± 8.2%). Statistically significant differences were detected between the measured percent thickness on day 3 and those measured on days 10 (^****^p < 0.0001), 15 (^**^p < 0.01), and 30 (^***^p < 0.001). Whereas no statistically significant differences were found comparing the thickness measured on days 10, 15, and 30 (Figure 2F).

#### Evaluation of the number of CD-68-positive cells (macrophages) over time

The number of CD-68-positive macrophages was calculated histomorphometrically per square millimeter. Three days after implantation, some CD-68-positive cells were found within the implantation bed in the test group (33.7 ± 12.5 cells/mm^2^), whereas a significantly higher number was found within the implantation bed in the control group (105.9 ± 16.3 cells/mm^2^; ••••*p* < 0.0001). The number of these cells increased by day 10 (185.9 ± 8.5 cells/mm^2^) in the test group. On the contrary, there was a rapid decrease in the number of CD-68-positive cells in the control group (19.6 ± 3.7 cells/mm^2^). At this time point, the highest number of macrophages was measured in the test group throughout the study period and was significantly higher than in the control group (••••*p* < 0.0001). By day 15, the number of macrophages was decreased in the test group (118.9 ± 8.5 cells/mm^2^) and in the control group (12.5 ± 4.2 cells/mm^2^). At this time point, the difference was still highly significant (*p* < 0.0001). Similar numbers of macrophages observed on day 15 were observed on day 30 in the respective test (109.8 ± 14.4 cells/mm^2^) and control groups (8.9 ± 2.9 cells/mm^2^). At this timepoint, significantly higher number of CD-68 positive cells was detected within the test group compared to the control group (••••*p* < 0.0001).

**Figure 5:**
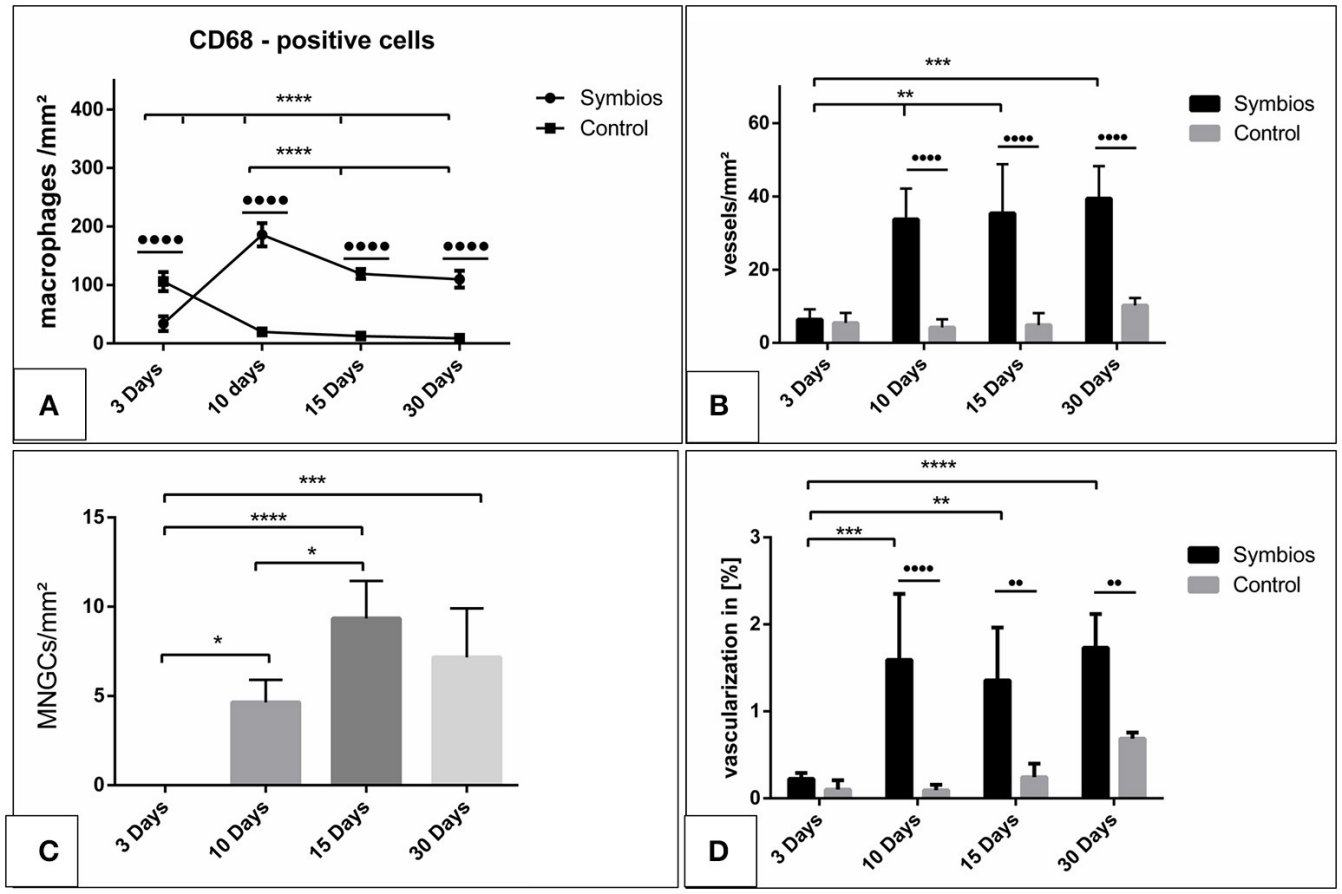
Histomorphometrical analysis. **(A)** The numbers of CD-68-positive cells (macrophages) per square millimeter over the time. **(B)** The vascularization pattern over the time in vessels per square millimeter in comparison to the control group. **(C)** The numbers of multinucleated giant cells (MNGCs) per square millimeter over the time. **(D)** The percent vascularization of the implantation bed over the time in comparison to the control group. (^*^^/•^*p* < 0.05; ^**^^/••^*p* < 0.01; ^***^^/•••^*p* < 0.001; and ^****^^/••••^*p* < 0.0001).

Within-group analyses in the test group showed that the increase in the number of macrophages from day 3 to 10 was highly significant (^****^p < 0.0001). Moreover, the decrease from day 10 to 15 showed a statistically significant difference (^***^p < 0.001). Additionally, the difference in macrophage number between day 10 and 30 was statistically highly significant (^****^p < 0.0001). Despite the decrease from day 10 onward, the numbers of macrophages on days 15 and 30 were higher. The difference was statistically highly significant when comparing the numbers between day 3 and day 15 (^****^p < 0.0001) and between day 3 and day 30 (^****^p < 0.0001). However, no statistically significant difference was observed in the macrophage numbers between days 15 and 30 (Figure 5A).

#### Evaluation of the number of multinucleated giant cells over time

No MNGCs were observed in the control group at any time point; therefore, it was not considered for the statistical analysis.

The number of the MNGCs was determined histomorphometrically per square millimeter. Three days after implantation, no MNGCs were found within the implantation bed of the biomaterial. After 10 days, a moderate number of MNGCs were present within the biomaterial implantation bed (4.6 ± 1.3 MNGCs/mm^2^). The number of the MNGCs increased significantly toward day 15 (9.3 ± 2.1 MNGCs/mm^2^). However, after 30 days, a slight decrease in the MNGC number was detected (7.2 ± 2.7 MNGCs/mm^2^) compared to the number of MNGCs at day 15.

Statistical analysis showed a significant difference in the MNGC number between days 3 and 10 (^*^p < 0.05). The number of MNGCs was significantly higher on day 15 than on day 3 (^****^p < 0.0001) and on day 10 (^*^p < 0.05). The slight decrease from day 15 to 30 showed no statistically significant difference. Moreover, no statistically significant difference was observed in the MNGC numbers between days 30 and 10. However, the number of MNGCs was significantly higher on day 30 than on day 3 (^***^p < 0.001). Generally, the MNGCs showed no TRAP expression. Therefore, no histomorphometrical analysis of the TRAP expression was performed (Figure 5C).

#### Evaluation of the vascularization pattern over time

The vascularization pattern was evaluated histomorphometrically in the biomaterial implantation bed as well as the sham operation group.

The vessel density on day 3 within the SB implantation bed (6.4 ± 2.8 vessels/mm^2^) and the control group (5.3 ± 2.7 vessels/mm^2^) showed comparable results. Thus, no statistically significant difference was revealed at this time point. Ten days after implantation, the SB implantation bed (33.7 ± 8.4 vessels/mm^2^) showed a higher vascularization rate than that on day 3. The difference was statistically significant (^**^p < 0.01). The vessel density within the control group increased only slightly on day (5.9 ± 2.2 vessels/mm^2^). Therefore, the vessel density within the SB group showed a significantly higher rate than that in the control group (••••*p* < 0.0001). After 15 days, the vessel density within the SB implantation bed was increased slightly (35.4 ± 13.4 vessels/mm^2^). No statistically significant difference was detected compared to day 10. However, the vessel density was significantly higher than that on day 3 (^**^p < 0.01). In addition, a minor increase in the vessel number was observed in the control group (6.3 ± 3.8 vessels/mm^2^). The SB group showed a significantly higher vessel number than the control group at this time point (••••*p* < 0.0001). On day 30, an increase in the vessel density within the SB group was observed (39.4 ± 8.8 vessels/mm^2^). However, no statistically significant difference was revealed between days 10 and 15. The number of vessels was significantly higher than the vessel density on day 3 (^***^p < 0.001). The vascularization of the control group showed an increase in the vessel density (10.2 ± 2.1 vessels/mm^2^). However, the SB group showed a significantly higher vessel density compared to the control group at this time point (••••*p* < 0.0001; Figure 5B).

The vascularization was quantified with respect to the implantation area in both the SB group and the control group. The percent vascularization increased over 30 days in both groups. On day 3, both groups showed comparable values (SB: 0.2 ± 0.07%; control: 0.15 ± 0.08%), and there was no statistically significant difference. The percent vascularization increased in the SB group significantly by day 10 (1.6 ± 0.06%). At this time point, the percent vascularization within the SB implantation bed was significantly higher than that in the control group (0.12 ± 0.06%; ••••*p* < 0.001). Subsequently, on day 15, the percent vascularization rate within the SB group (1.3 ± 0.6%) showed a slight decrease compared to the previous time point. However, the vascularization was significantly higher than that on day 3 (^**^p < 0.01), whereas no statistically significant difference was detected compared to the vascularization on day 10. The difference between the vascularization in the SB group and in the control group (0.2 ± 0.1%) showed a significantly higher vascularization in the SB group at this time point (••*p* < 0.001). Finally, after 30 days, the percent vascularization increased in the SB group (1.7 ± 0.3%). This value was significantly higher than the percent vascularization on day 3 (^****^p < 0.0001). In contrast, no statistically significant differences were evident compared to the percent vascularization on days 15 or 10. The percent vascularization within the control group (0.7 ± 0.07%) increased compared to the previous time point. Nevertheless, the percent vascularization was significantly higher in the SB group than in the control group at this time point (••*p* < 0.01; Figure 5D).

**Figure 6:**
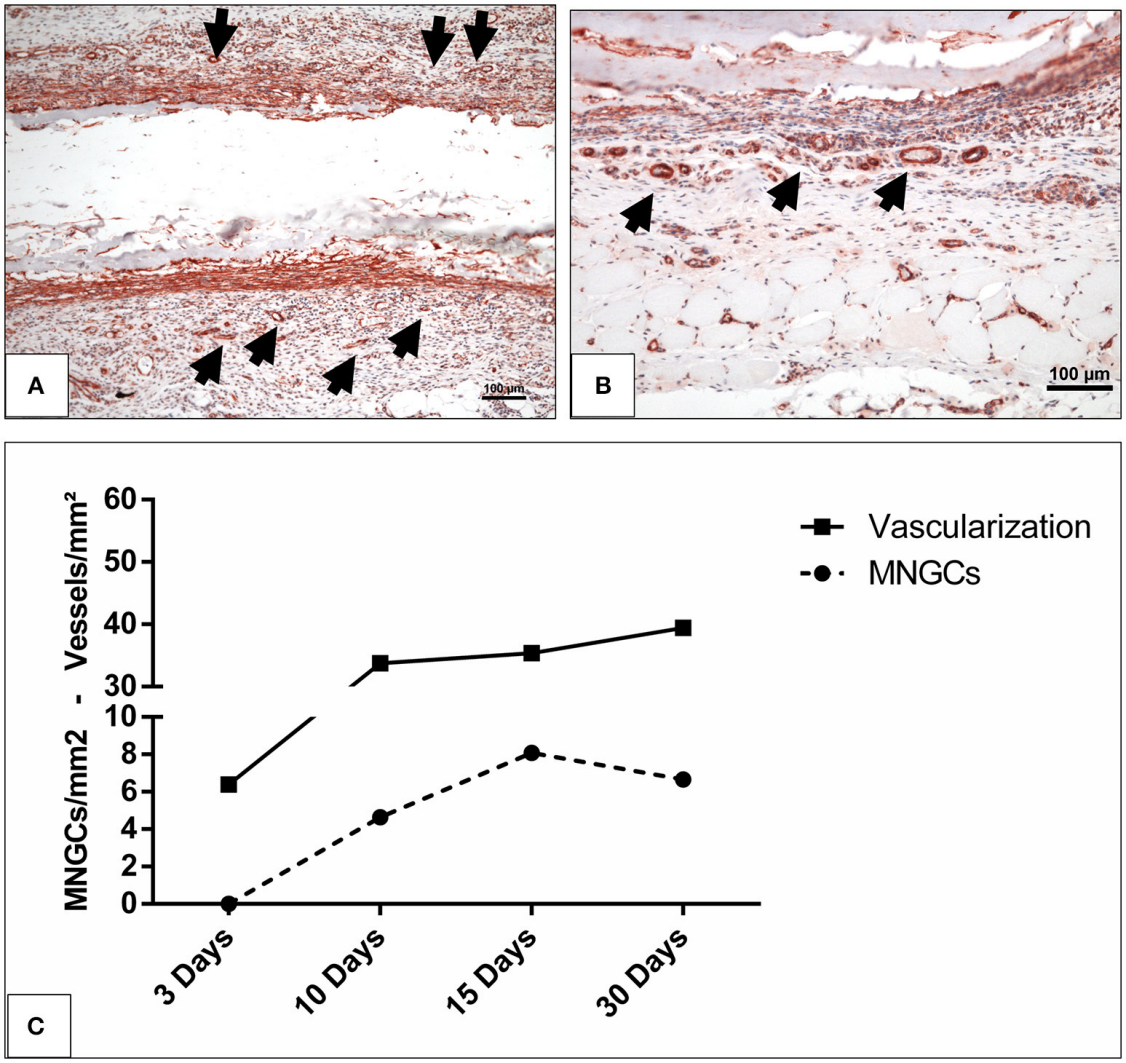
The vascularization pattern **(A)** on day 10 and **(B)** on day 15 highlighted with immunohistochemical staining of SMA in x200 magnification. Black arrow heads, vessels; scale bar, 100 μm. **(C)** The correlation between the induced MNGCs and the vascularization in the test group.

Additionally, similar kinetics were detected in the vascularization and induction of MNGCs. The vascularization pattern increased corresponding to the increasing number of the induced MNGCs over time (Figures 6A–C).

## Discussion

Numerous collagen-based membranes are available as naturally derived xenogeneic membranes that should meet the defined requirements to serve as a scaffold and maintain integrity for a suitable time period. However, the naturally derived xenogeneic collagen-based biomaterials must undergo different purification and processing procedures that influence their native structure. In this context, the cellular reaction toward different membranes is primarily related to the membrane-specific physicochemical properties (Dadsetan et al., 2004; Ghanaati et al., 2012).

The *in vivo* tissue response toward a novel collagen-based biomaterial SB that is derived from bovine achilles tendon showed that SB initially induced a mononuclear cell-based reaction on day 3. At this time point, single mononuclear cells had already infiltrated the membrane. Immunohistochemical staining showed that some of the mononuclear cells were CD-68-positive, representing the presence of macrophages on the SB surface. The mid-term cellular reaction on day 10 was characterized by a high number of macrophages (CD-68-positive) on the membrane surface, reflecting a significant increase in their number compared the number of macrophages observed on day 3. Additionally, some MNGCs were found on the biomaterial surface at this time point. The induction of MNGCs was accompanied by significantly increased vascularization. However, the localization of the mononuclear cells was rather triggered within the central region, whereas MNGCs were only located on the membrane surface (Figure 3).

MNGCs the potential to express vascular endothelial growth factor, which may explain the enhanced vascularization within the implantation bed after their formation (Moens et al., 2014). This phenomenon was previously observed within the implantation bed of different biomaterials including synthetic and xenogeneic bone substitute materials and collagen based-membranes (Ghanaati et al., 2010a; Barbeck et al., 2015b,c). By day 15, a course change was detected in the number of the macrophages and MNGCs (Figure 4). The macrophage number decreased significantly in comparison to that observed on day 10. Simultaneously, the number of the MNGCs increased significantly compared to that observed on day 10. Macrophages are precursors cells of MNGCs (Anderson et al., 2008; Chen et al., 2010). Their interaction with the biomaterial surface and the inflammatory microenvironment around the implanted biomaterial is essential for the fusion and formation of MNGCs, which possess an enhanced oxidative capacity (Enelow et al., 1992; McNally and Anderson, 2002; Chang et al., 2009). This process was previously described as frustrated phagocytosis. Thus, after the frustrated attempts of macrophages to degrade the biomaterial, they fuse to form MNGCs (Xia and Triffitt, 2006; MacLauchlan et al., 2009). Accordingly, the dynamic changes observed here in the numbers of macrophages and MNGCs might be due to their fusion and the process of MNGC formation. At the last observation time point, neither the macrophages nor the MNGCs showed a significant change in the number of cells. These findings were similar to the rate of vascularization, which showed a stable level from day 10 onwards. All the parameters presented here, including the formation of MNGCs and enhanced vascularization, are circumstantial evidence of a foreign body reaction (Mcnally and Anderson, 2015). In this sense, it is questionable whether an exuberant vascularization is required for the regeneration process. It was previously shown that a mild vascularization that is derived by mononuclear, similar to the physiological vascularization pattern is sufficient for membrane integration *in vivo* (Ghanaati, 2012).

The present results showed that the occurrence of MNGCs is induced by the implanted biomaterial, thus no MNGCs were observed within the control group, which simulated physiological wound healing. Previous assumptions questioned whether the formation of MNGCs is a physiological reaction related to biomaterial resorption or might be associated with collagen-based biomaterials harvested from a specific compartment (Barbeck et al., 2015b). However, MNGCs were observed within the implantation bed of different biomaterials including collagen-based biomaterial (Barbeck et al., 2015b) and silk-based polymers (Ghanaati et al., 2010c), as well as different synthetic and xenogeneic bone substitute materials (Ghanaati et al., 2010b). The biomaterial-induced MNGCs show common characteristics with pathological MNGCs (Langerhans' giant cells) that exist in sarcoidosis and tuberculosis (Al-Maawi et al., 2017). These are not only morphological characteristics in terms of the number of nuclei but also in the expressed surface proteins such as CD-68, Integrin ß 1/2 and HLA-DR (Al-Maawi et al., 2017). In this sense, the question arises as to whether the presence of MNGCs within the implantation bed of clinically applicable biomaterials is an acceptable reaction or rather an adverse reaction after chronic inflammation.

It is noteworthy that collagen-based biomaterials, such as non-cross-linked bilayer collagen matrix from porcine skin and peritoneum, induced solely mononuclear cells and maintained their native structure over a period of 60 days. This collagen matrix allowed slow penetration into the biomaterial superficial layer but served as a functional barrier within its central region and was integrated within the host tissue (Ghanaati et al., 2011).

Additionally, in the present study MNGCs were only localized on the surface of the biomaterial and did not enter the biomaterial body, while the mononuclear cells migrated into the membrane body and were integrated after 30 days. Therefore, the vascularization was enhanced at the peri-implantation region, but no vessels were observed within the membrane central region in terms of transmembraneous vascularization. The occurrence of inflammatory responses with two different outcomes, i.e., mononuclear cells vs. MNGCs, is noteworthy. This specific inflammatory pattern may be beneficial to recruit vessels to the implantation region without manipulating the membrane structure. This may be related to the specific architecture of the biomaterial that includes pores within the membrane body, which allowed the mononuclear cells to migrate into the center region and integrate within the biomaterial so that the macrophages were not over-accumulated at the surface, resulting in a high number of MNGCs. In addition, despite the presence of MNGCs, the SB maintained its integrity over 30 days and allowed only mononuclear cells to enter its central region while resisting the MNGCs (Figure 3). Thereby, no signs of breakdown or disintegration in terms of loss of the initial structure, were detected during the evaluation period. Additionally, within the observation period of this study, the biomaterial did not show typical signs of encapsulation as it was previously found in non-resorbable biomaterials (Ghanaati, 2012) Furthermore, it is interesting to further elucidate whether the presence of MNGCs will lead to a classical foreign body reaction and encapsulation of the biomaterial or whether these cells could be involved within the resorption process of the biomaterial.

In contrast, other non-cross-linked, collagen-based biomaterials that induced MNGCs underwent disintegration after a clear backdown leading to their disintegration (Barbeck et al., 2015b). In that case, the loss of integrity allowed premature ingrowth of the peri-implantation connective tissue into the membrane body including MNGC- and vessel-rich granulation tissue (Barbeck et al., 2015b). This phenomenon was not observed in this study during the evaluation period. Within the limitations of this study, it is presumable that the membrane may experience disintegration after 60 days or more.

Another naturally derived biomaterial of silk fibroin underwent disintegration after the induction of MNGCs. The silk fibroin showed transmembraneous vascularization and a loss of integrity after 60 days (Ghanaati, 2012). However, most of the induced MNGCs in silk fibroin expressed TRAP, which might be a sign for their pro-inflammatory activity (Ghanaati, 2012). On the other hand, the present results showed mostly no TRAP-positive MNGCs, which might represent a different MNGC type that did not contribute to the disintegration of the biomaterial. Another parameter may be the harvesting compartment of the SB, i.e., bovine achilles tendon. This may include a different collagen quality that does not evoke a severe pro-inflammatory reaction.

Furthermore, different processing and purification methods have an impact on changing the surface characteristics of the native collagen, which leads to different tissue responses (Jones et al., 2004). This may influence the cellular activation and thus the macrophage polarization and expression pattern, which possibly influence their fusion to MNGCs (Jones et al., 2004; Kajahn et al., 2012). However, further studies are needed to determine the interaction between differently activated macrophages and the formation of MNGCs for a better understanding of the mononuclear cells and multinucleated giant cell activation and polarization.

The *ex vivo* results of the interaction between SB and the liquid PRF showed that the membrane was infiltrated by liquid PRF including leukocytes and platelets. Therefore, the membrane absorbed the liquid PRF and allowed cellular invasion during the early time period (Figure 1). However, *in vivo* results after 3 days showed some cellular infiltration intro the superficial layer of the membrane, whereas the central region was still free of murine cells of the peri-implantation region. This occurs because of the difference between the qualities of subcutaneous tissue and the liquid PRF. Thereby, *in vivo* cellular infiltration require longer time period until cells migrate from the extracellular matrix of the peri-implantation region into the membrane compartment. Nevertheless, cellular infiltration was then reached after an initial time period of 10–15 days *in vivo*.

Accordingly, the *in vivo* thickness measurements showed that the SB thickness increased significantly from day 3 to day 10 and then maintained the thickness level over 30 days. This occurred because the SB allowed cells and connective tissue to enter the membrane body. The specific structure and interfibrillar compartments allowed the membrane to include the host cells and connective tissue and thus increase in thickness without undergoing a breakdown or disintegration (Figure 3). Recently, the application of this method for the evaluation of a sugar cross linked porcine derived collagen membrane showed that the membrane was occlusive to the fibrin and cells of PRF (Chia-Lai et al., 2017). These results were in correlation to the *in vivo* evaluation using subcutaneous implantation. This frequent agreement in the *ex vivo* and *in vivo* results make liquid PRF a potential tool to investigate the membrane absorbance capacity and provide information about the *in vivo* cellular reaction while avoiding animal experiments. Recent studies used blood serum and plasma proteins to investigate the biomaterial surface absorption capacity (Nguyen et al., 2016). In the present study, a more complex system was used i.e., liquid PRF, which includes not only plasma proteins but also cells (Platelets and leukocytes). Thereby, the focus of the present study was placed on the cellular infiltration of the collagen-based membrane by the cells and the formation of the fibrin network within the membrane pores. Additionally, to further elucidate the mechanisms of protein absorbance and the interaction between collagen and PRF, further methods are required such as atomic force microscopy. These aspects are further topics of our research group and are presently under investigation to elucidate the capacity of different collagen based biomaterials to incorporate PRF with special focus on different plasma proteins respecting the competitive protein exchange and the vroman effect (Hirsh et al., 2013).

In addition, the specific porous structure and the ability to include the host connective tissue might be a reason for the maintained integrity after inducing a foreign body reaction by MNGCs. It might be that including the host cells within the membrane is favorable for the regeneration process to serve as a scaffold to promote guided tissue regeneration. In an animal study using a tooth dehiscence model for periodontological regeneration, a collagen biomaterial of bovine achilles origin showed comparable results to a non-cross-linked, porcine-derived collagen membrane, which led to successful tissue regeneration (Behfarnia et al., 2012). Furthermore, a clinical study showed that bovine-derived collagen membranes are suitable for successful root coverage (Schlee et al., 2012).

In summary, the present findings raise the need for further research to characterize the types of biomaterial-related MNGCs and whether they should be accepted as a biomaterial-related cellular reaction or considered as an adverse reaction following chronic inflammation. These findings are clinically highly interesting to evaluate the clinical suitability of different biomaterials and define suitable indications with respect to the biomaterial physicochemical properties.

## Conclusion

The present study evaluated the cellular reaction toward a novel collagen membrane derived from bovine achilles tendon. The tissue response showed an initial reaction of mononuclear cells followed by the formation of MNGCs from day 10 onwards, whereas no MNGCs were detected within the control group that mimicked physiological wound healing. The presence of these cells was accompanied by a reduction in the CD-68-positive cell number (macrophages), indicating their fusion to form MNGCs, which were only localized on the membrane surface. Along with the enhanced MNGC number, the vascularization of the peri-implantation area increased significantly. No transmembraneous vascularization was found within the membrane body, and only mononuclear cells were able to migrate. These characteristics refer to a foreign body reaction toward the biomaterial surface. Thereby, the role of the MNGCs induced by this biomaterial requires further investigation.

*Ex vivo* and *in vivo* experiments showed that the biomaterial allows protein absorbance and mononuclear cells to migrate into its central region. These findings were observed along with the *in vivo* increase in thickness reflecting the membrane capacity to incorporate the host cells and connective tissue and form a scaffold without undergoing any signs of breakdown or disintegration. These findings raise the question of whether the formation of MNGCs should be accepted as a biomaterial-related reaction or considered as an adverse reaction.

## Author contributions

SA-M: Immunohistochemical analysis, data acquisition, statistical analysis, manuscript preparation, manuscript editing, literature research; CV: Histological preparation, Data acquisition, histomorphometric analysis, statistical analysis, literature research, *Ex vivo* study part; AO: Animal surgery, data acquisition, histology; TZ: *Ex vivo* study part, literature research, data analysis; RS: Definition of intellectual content, manuscript review; CK: Definition of intellectual content, manuscript review; SG: Study concept, study design, definition of intellectual content, manuscript review, manuscript editing.

### Conflict of interest statement

The authors declare that the research was conducted in the absence of any commercial or financial relationships that could be construed as a potential conflict of interest.
